# Short-Term Metformin Treatment Enriches *Bacteroides dorei* in an Obese Liver Steatosis Zucker Rat Model

**DOI:** 10.3389/fmicb.2022.834776

**Published:** 2022-03-30

**Authors:** Michael S. Robeson, Kanishka Manna, Christopher Randolph, Stephanie Byrum, Reza Hakkak

**Affiliations:** ^1^Department of Biomedical Informatics, University of Arkansas for Medical Sciences, Little Rock, AR, United States; ^2^Department of Biochemistry and Molecular Biology, University of Arkansas for Medical Sciences, Little Rock, AR, United States; ^3^Arkansas Children’s Research Institute, Little Rock, AR, United States; ^4^Department of Dietetics and Nutrition, University of Arkansas for Medical Sciences, Little Rock, AR, United States; ^5^Department of Pediatrics, University of Arkansas for Medical Sciences, Little Rock, AR, United States

**Keywords:** obesity, metformin, *Bacteroides dorei*, Zucker rat, gut microbiota

## Abstract

Obesity is the leading cause of health-related diseases in the United States and World. Previously, we reported that obesity can change gut microbiota using the Zucker rat model. Metformin is an oral anti-hyperglycemic agent approved by the FDA to treat type 2 diabetes (T2D) in adults and children older than 10 years of age. The correlation of short-term metformin treatment and specific alterations to the gut microbiota in obese models is less known. Short-term metformin has been shown to reduce liver steatosis. Here we investigate the effects of short-term metformin treatment on population of gut microbiota profile in an obese rat model. Five week old obese (*n* = 12) female Zucker rats after 1 week of acclimation, received AIN-93 G diet for 8 weeks and then rats were randomly assigned into two groups (6 rats/group): (1) obese without metformin (ObC), or (2) obese with metformin (ObMet). Metformin was mixed with AIN-93G diet at 1,000 mg/kg of diet. Rats were weighed twice per week. All rats were sacrificed at the end of metformin treatment at 10 weeks and fecal samples were collected and kept at −80°C. Total microbial DNA was collected directly from the fecal samples used for shotgun-metagenomics sequencing and subsequently analyzed using MetaPlAn and HUMAnN. After stringent data filtering and quality control we found significant differences (*p* = 0.0007) in beta diversity (Aitchison distances) between the ObC vs. ObMet groups. Supervised and unsupervised analysis of the log-ratios *Bacteroides dorei* and *B. massiliensis* vs. all other *Bacteroides* spp., revealed that *B. dorei* and *B. massiliensis* were enriched in the ObMet group, while the remaining *Bacteroides* spp. where enriched in the ObC group (*p* = 0.002). The contributional diversity of pathways is also significantly associated by treatment group (*p* = 0.008). In summary, in the obese Zucker rat model, short-term metformin treatment changes the gut microbiota profile, particularly altering the composition *Bacteroides* spp. between ObC and ObMet.

## Introduction

Obesity has been an epidemic in the United States (US) and the rate of adult obesity continues to grow. Data from the Centers for Disease Control and Prevention (CDC) indicated that more than one-third of United States adults are obese (Ogden et al., 2014). However, recent data from CDC indicate that In 2017–2018, the age-adjusted prevalence of obesity in adults was 42.4%, and there were no significant differences between men and women among all adults or by age group. The age-adjusted prevalence of severe obesity in adults was 9.2% and was higher in women than in men (Flegal et al., 2016). Obesity is associated with several health problems such as type 2 diabetes, cardiovascular disease, liver disease and certain types of cancers (Hakkak et al., 2012). Childhood obesity has more than doubled in children, and obesity has quadrupled in adolescents in the past 30 years (Angulo, 2007; Ogden et al., 2008; Fryar et al., 2021). In the United States, the prevalence of obesity is 19.3% and affects about 14.4 million children and adolescents. Obesity prevalence was 13.4% among 2- to 5-year-olds, 20.3% among 6- to 11-year-olds, and 21.2% among 12- to 19-year-olds (Fryar et al., 2021). Obesity is often associated with an increased risk of non-alcoholic fatty liver disease (NAFLD), in which liver steatosis is commonly observed (Fabbrini et al., 2010).

Non-alcoholic fatty liver disease is the leading cause of liver disease in adolescents in the United States and world, and the risk has increased with the rise of obesity (Lazo et al., 2013; Welsh et al., 2013). Data from our animal studies using the obese Zucker (*fa/fa*) rat model reported that obesity increases fatty liver (steatosis) and that obese Zucker rats can develop fatty liver by the starting age of 8 weeks (Hakkak et al., 2018, 2021).

The effects of obesity on composition and metabolic activity of the intestinal microbiota is an active area of study (Ley et al., 2005; Tremaroli and Bäckhed, 2012; Devaraj et al., 2013; Harsch and Konturek, 2018; Pascale et al., 2019; Cao et al., 2020; Zhang and Hu, 2020). Several studies have identified some differences between the microbiota populations in lean and obese subjects (Tremaroli and Bäckhed, 2012). Mice homozygous for the leptin receptor mutation that results in the development of obesity show a reduction in *Bacteroidetes* and an increase in *Firmicutes* compared to their wild-type siblings when fed the same diet. This effect is not limited to animals with a genetic predisposition to obesity. Diet-induced obesity is also linked to changes in the intestinal microbiota in mice (Turnbaugh et al., 2008). This connection between adiposity and the gut microbial ecology appears to apply to humans as well (Turnbaugh et al., 2009). It is clear that both community composition and discrete bacterial species can exert either pathogenic effects that encourage disease development or probiotic effects that maintain health status (Devaraj et al., 2013).

We recently reported the effects of obesity on gut microbiota using a Zucker rat model *via* amplicon sequencing of the 16S rRNA gene (Hakkak et al., 2017). Several groups of bacteria were differentially abundant between lean and obese rats after 60 days. Furthermore, we found that principal coordinate analysis (PCoA) plots of beta diversity, and LEfSe analysis (Segata et al., 2011) suggested differences in intestinal microbiota populations associated with both time point, and lean or obese status, within the Zucker rat model for obesity (Hakkak et al., 2017). The scientific community emphasizes the need to investigate the effects of metformin in conjunction with gut microbiota (McCreight et al., 2016; Rodriguez et al., 2018; Pascale et al., 2019; Zhang and Hu, 2020). The gut microbiota contains a diverse population of obligate and facultative anaerobic microorganisms that contribute a broad range of metabolic activities. These microorganisms usually exist in a symbiotic relationship with the host and are important in the digestion of dietary components and the metabolism of nutrients and drugs (Ley et al., 2006; Vázquez-Baeza et al., 2018; Weersma et al., 2020). The specific population of organisms comprising the intestinal microbiota in an individual is relatively stable under normal conditions, but several factors, such as diet, disease state, antimicrobial use, etc., can cause changes in the distribution of different bacterial groups (Turnbaugh et al., 2009; Tagliabue and Elli, 2013). These population changes can affect the metabolic capabilities of the total microbiota population, which can affect the health of the host (Rodriguez et al., 2018; Vázquez-Baeza et al., 2018; Zimmermann et al., 2019).

Members of the genus of *Bacteroides* (e.g., *B. vulgatus* and *B. dorei*) are mostly gram-negative anaerobic organisms. *Bacteroides* spp. are often characterized as a predominant gut bacterial species (Wexler and Goodman, 2017). *Bacteroides dorei*, was recently isolated and distinguished from *Bacteroides vulgatus* (Pedersen et al., 2013). Prior to this, both species were difficult to disambiguate until the advent of 16S rRNA gene amplicon sequencing methods (Pedersen et al., 2013). Although very short-read high-throughput sequencing of the 16S rRNA gene is unable to differentiate among the two taxa, success can be achieved by targeting the longer V3V4 region of the 16S rRNA gene (Davis-Richardson et al., 2014). This potentially explains seemingly contradictory results across ampicon-based microbiome surveys regarding the identity of *Bacteroides* spp. Prior studies have shown that these organisms might improve the enteric environment and reduce bacterial lipopolysaccharide (LPS) production (Yoshida et al., 2018). LPS is confirmed as a potent inducer of hepatic inflammation like NAFLD in obese subjects (Fukunishi et al., 2014).

Metformin is an oral anti-hyperglycemic agent approved by the FDA to treat type 2 diabetes (T2D) in adults and children older than 10 years of age. Several clinical trials have identified modest improvements following metformin treatment in insulin sensitivity in obese children with normal glucose tolerance (Srinivasan et al., 2006; Burgert et al., 2008; Love-Osborne et al., 2008; Brufani et al., 2013; McDonagh et al., 2014; Lentferink et al., 2018; Raman and Foster, 2021), as well as a decrease in the BMI of obese adolescents (Wilson et al., 2010). In addition, metformin appears to improve lipid profiles in obese children (Kay et al., 2001; Atabek and Pirgon, 2008). Furthermore, metformin is not only used for the treatment of diabetes, but also for various other diseases including cancer, cardiovascular diseases, and liver steatosis (Lin et al., 2000; Foretz et al., 2014; Madsen et al., 2015; Wang et al., 2016; Fujita and Inagaki, 2017; Lv and Guo, 2020; Hakkak et al., 2021).

Our prior research has also shown that short-term dietary effects can be observed when obese Zucker rats are fed a diet of soy protein with high isoflavones that can protect against liver steatosis. Although these rats have gained more weight compared to obese casein-fed rats, they had lower liver steatosis and contained lower blood serum levels aspartate aminotransferase AST, alanine aminotransferase (Hakkak et al., 2018). Similar results were observed for obese Zucker rats fed an AIN-93 G diet during metformin treatment (Hakkak et al., 2021). Prior research within murine models and human subjects has shown that short-term metformin treatment was sufficient to reduce liver steatosis (Lin et al., 2000; Madsen et al., 2015; Wang et al., 2016; Hakkak et al., 2021).

Although various mechanisms by which metformin acts are still being investigated, the last decade of research has led to some insightful discoveries on how the gut microbiome responds to and contributes to the altered metabolic landscape driven by metformin treatment, as reviewed by Sanz et al. (2015), McCreight et al. (2016), and Pascale et al. (2019). The ability of the microbiome to affect many other therapeutic treatments is well known and of increasing interest (Vázquez-Baeza et al., 2018; Zimmermann et al., 2019). Investigations on the effects of metformin on the gut microbiota have shown increasing evidence that a key factor of metformin action involves the gut microbiome (Pastor-Villaescusa et al., 2016; de la Cuesta-Zuluaga et al., 2017; Pascale et al., 2019). The effects of metformin on murine models through the use of high-fat diet-induced obesity (Zhang and Hu, 2020) revealed that metformin had significant effects and changes on the composition of the gut microbiota (Lee and Ko, 2014; Shin et al., 2014; Cao et al., 2020). However, the correlation of short-term metformin treatment and specific alterations to the gut microbiota in obese models and liver steatosis is less known.

We have previously shown that short-term (10 weeks) metformin treatment is a useful model for early adolescent obesity related diseases, using obese Zucker rat model (Hakkak et al., 2021). In this model, we were able to show that this short-term metformin treatment can protect against NAFLD. However, the possible mechanisms of this protection is less known. Herein we extend our investigations of the gut microbiome by focusing on the effects of short-term metformin treatment on obese Zucker rats using shotgun metagenomics to better resolve the species and strain-level identification of microbial taxa (Hong et al., 2009; Jovel et al., 2016; Wasimuddin et al., 2020).

## Materials and Methods

### Experimental Design

All animal care and procedures were approved by the University of Arkansas for Medical Sciences/Arkansas Children’s Research Institute Institutional Animal Care and Use Committee and adhered to the institutional policies and procedures. The guidelines of the United States Department of Agriculture (USDA) Animal Welfare Act were followed to ensure that the care and use of animals were appropriate and humane.

### Sampling and Storage

A total of 12 five-week-old female obese (*fa/fa*) Zucker rats were purchased from Envigo, (Indianapolis, IN, United States), as they are sexually mature by this age (Sengupta, 2013). Female obese Zucker (*fa/fa*) rats are often used for non-diabetic obesity studies because they are highly resistant to developing diabetes unless fed a high fat diet (Corsetti et al., 2000; Gustavsson et al., 2011), whereby we can investigate obesity and liver steatosis without the confounding effects of a diabetic phenotype. We have also shown that both obese male and female rats will develop obesity and liver steatosis at the same rates and that there is no difference on between both sexes (Hakkak et al., 2012, 2015, 2018). Rats were housed in an Association for Assessment and Accreditation of Laboratory Animal Care approved animal facility that is registered with the USDA and has a fully approved Letter of Assurance on file with the Office of Laboratory Animal Welfare of the National Institutes of Health. Rats were housed one per cage in 12-h light-dark cycles and had *ad libitum* access to feed and water. After 1 week of acclimation (age 42 days), rats had *ad libitum* access to water on semi-purified AIN-93G diet (Envigo, Indianapolis, IN) for 8 weeks to mimic obese adolescents (Hakkak et al., 2012, 2015, 2018). Rats were weighed twice weekly. After 8 weeks on AIN 93-G diet, obese rats were randomly assigned into two groups (6 rats/group): (1) obese without metformin (ObC), or (2) obese with metformin (ObMet), and maintained for 10 weeks. Metformin was mixed with AIN-93G diet at 1,000 mg/kg of diet (Envigo, Indianapolis, IN, United States). We used a modified formula of the Reagan-Shaw approach for obesity to calculate the maximum dose of metformin in the proposed experiment (Food and Drug Administration, 2005). All rats were euthanized after treatment in the 10th week. Fecal samples were collected over a 12-h period a day before the metformin treatment diet and at the end of the experiment. Fecal samples were stored at −80°C until analysis.

### DNA Extraction and Sequencing

Total microbial DNA was collected directly from the fecal samples using a PowerSoil ^®^ DNA isolation kit (MoBio Laboratories, Inc., Carlsbad, CA, United States). Isolated DNA was used for shotgun-metagenomics data collection using Illumina NextSeq 500 (Supplementary Figure 1). Minimum Information about a Metagenomic Sequence (MIMS) compliant data (Field et al., 2007, 2008) are available from the National Center for Biotechnology Information Sequence Read Archive, under BioProject PRJNA770726.

### Metagenomic Sequencing and Analyses

Shotgun metagenomic reads were first processed through the metaWRAP (Uritskiy et al., 2018) read_qc module which wraps FastQC (Andrews, 2010), Trim Galore (Martin, 2011; Krueger, 2015), and BMTagger (Rotmistrovsky and Agarwala, 2017), to filter and trim low-quality reads, and remove potential mammalian host sequences downloaded from GenBank (Benson et al., 2005), i.e., human (*Homo sapiens*; hg38), mouse (*Mus musculus*; mm10), rat (*Rattus norvegicus*; rn6), and pig (*Sus scrofa*; 10.2). The resulting reads were then processed *via* MetaPhlAn and HUMAnN *via* the bioBakery suite (Beghini et al., 2020) to determine microbial taxonomy and functional potential. Data preparation, as well as unsupervised and supervised microbial compositional analysis were performed using DEICODE (Martino et al., 2019), songbird (Morton et al., 2019), Qurro (Fedarko et al., 2020) and Emperor (Vázquez-Baeza et al., 2013; Supplementary Figure 2). The data were formatted for these analyses as outlined in Baker et al. (2021).

A taxonomic abundance table (feature-table) was generated from MetaPhlAn and subsequently filtered to contain only those bacteria that were identified at the species-level, and which were present in at least 50% of the samples (6 of 12), and contained at least 10,000 reads. This helps avoid spurious log ratios, as reviewed in Baker et al. (2021). Beta-diversity was calculated with DEICODE, which uses Robust Aitchison PCA, and visualized *via* Emperor. DEICODE was run with the following settings: min-sample-count 500, min-feature-count 1000, min-feature-frequency 0, max-iterations 10. Beta-diversity significance testing was performed through PERMANOVA. Upon visual inspection of the PCA plot, the feature-loadings of Axis 1 (which appeared to separate the control and metformin treatment groups), were visualized in Qurro. Either log-ratios of specific microbes (e.g., *Bacteroides* spp.), or those ranked above 0 and those equal or less than 0, were exported for *post-hoc* significance testing to determine microbiota associated with treatment group separation. This comprised our unsupervised analysis of microbial taxa.

The same taxonomy table was then analyzed *via* Songbird to rank microbial species that are associated with our metformin treatment through the use of reference frames. The following parameters were used: batch-size 3, num-random-test-examples 3, learning-rate 0.0001, epochs 50000, differential-prior 0.5, min-feature-count 6, summary-interval 1. This comprised our supervised analysis of microbial taxa.

A functional pathway table was generated with HUMAnN, which contains the functional potential of each metagenome sample. This data was analyzed similarly as the MetaPhlAn data above except in this case DEICODE was used to ordinate the samples with respect to functional pathway composition as they relate to the treatment groups, while Songbird was used to rank the pathways themselves as they relate to metformin treatment. The HUMAnN pathway table was filtered to keep pathways that were present in at least 50% of the samples (6 of 12) and contained at least 1,000 reads. The settings used for DEICODE: min-sample-count 500, min-feature-count 1000, min-feature-frequency 0, max-iterations 10. The setting used for Songbird: batch-size 3, num-random-test-examples 3, learning-rate 0.0001, epochs 100,000, differential-prior 0.5, min-feature-count 6, summary-interval 1.

## Results

### Body Weight

The final body weights for ObC vs. ObMet groups was 597.5 ± 41.4 g vs. 573.1 ± 48.1 g, respectively and not significantly different (*P* = 0.20).

### Microbial Diversity

**FIGURE 1:**
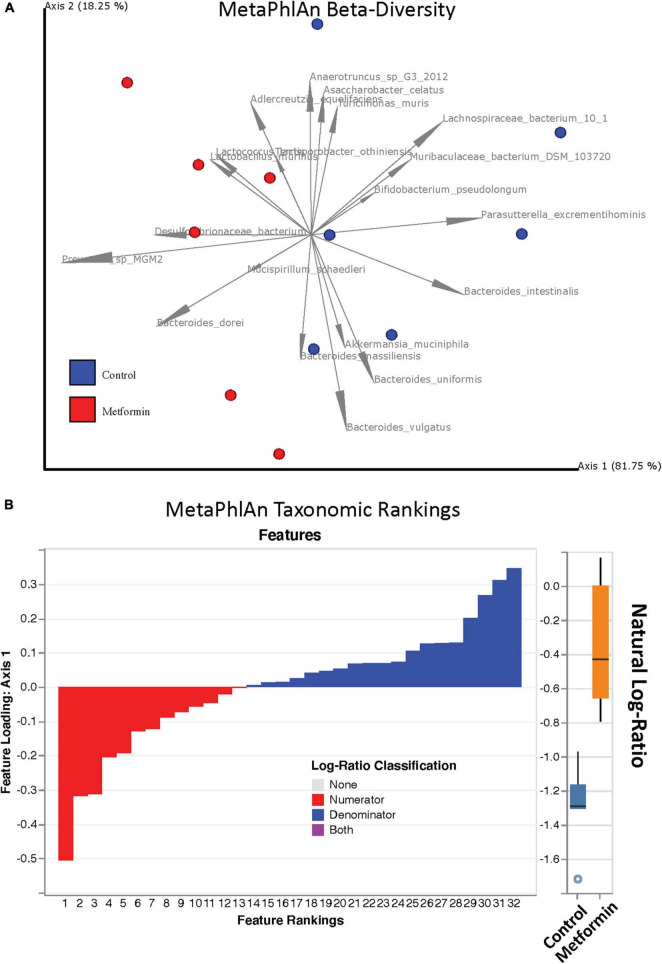
**(A)** Robust Aitchison PCA plot of metagenome samples processed through MetaPhlAn 3 and DEICODE and visualized with qurro. Bacterial species not present in at least 50% of samples were removed from the analysis. Separation of Control and Metformin treated groups was significant (*p*-value 0.002). **(B)** Corresponding log-ratio rankings for individual bacterial taxa oriented to Axis 1 (right). Numerator (red) / Denominator (blue) ratios of these taxa were computed to generate a box-whisker plot (left), *p*-value 0.0025. These results were corroborated with songbird analyses.

**FIGURE 2:**
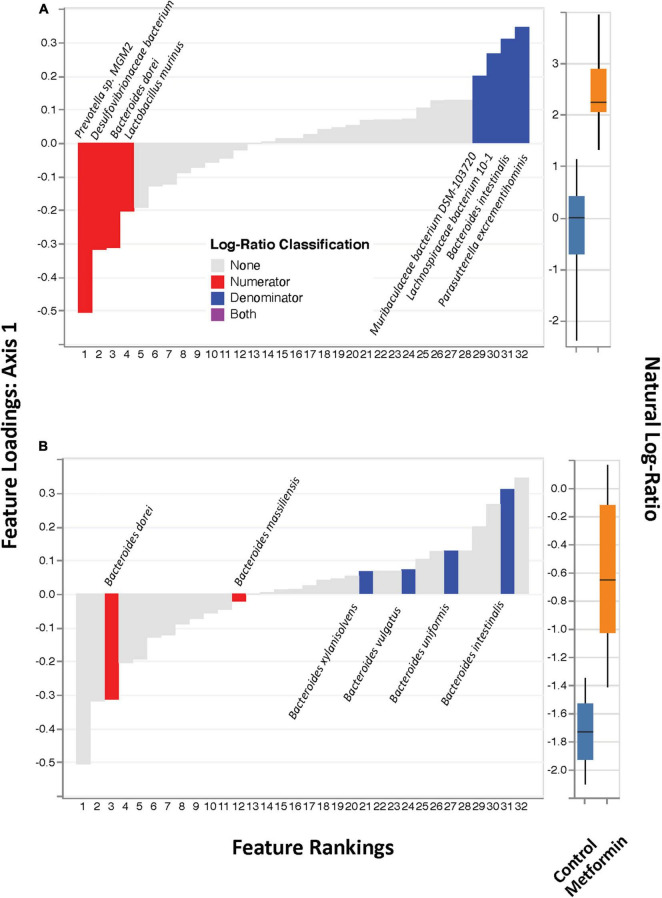
DEICODE feature rankings. **(A)** Same plot as in Figure 1, with only the top-feature rankings selected (left), and their ratios plotted as a box-whisker-plot (right). **(B)** Only *Bacteroides* spp. selected (left) and their ratios plotted as box-whisker plot (right).

No significant differences in alpha diversity were found between the control and metformin treated groups. However, significant differences in beta diversity were observed between these groups, as measured by Bray-Curtis (*p* < 0.011) and DEICODE (Figure 1; *p* < 0.002), but not Jaccard. DEICODE was further used as an unsupervised approach to detect differentially abundant ratios of microbiota. The log-ratio rankings of individual bacterial taxa were found to be significantly different (*p* < 0.0025) between the control and metformin groups after *post-hoc* analysis (Figure 1). This signal was still present when either the top feature-rankings were selected (Figure 2A), or only the feature rankings of *Bacteroides* taxa (Figure 2B). The most notable finding is the differential ratios of *Bacteroides* species present in each of the treatment groups, i.e., increased *B. dorei* and *B. massiliensis* (numerator taxa) in the metformin group relative to the control group, while the opposite is observed for (denominator taxa) *B. xylanisolvens*, *B. vulgatus*, *B. uniformis*, and *B. intestinalis* (Figure 2B). The above patterns were also observed through a supervised approach with Songbird (Supplementary Figure 3).

**FIGURE 3:**
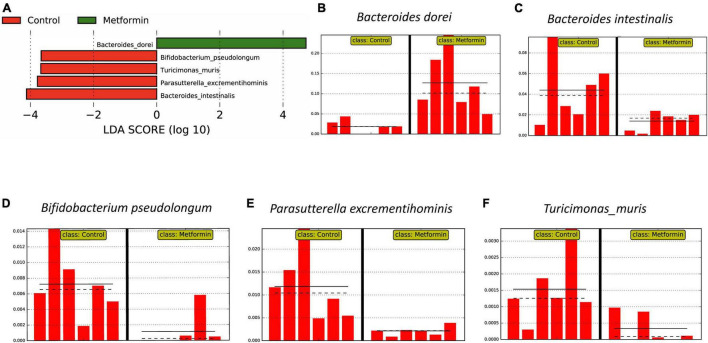
Differentially abundant bacterial taxa as determined by LEfSe analysis on MetaPhlAn 3 output. **(A)** Linear discriminant analysis (LDA) score plot of differential taxa associated with the Control and Metformin groups. Differential feature plots for the respective taxa are shown across samples: **(B)** *Bacteroides dorei*, **(C)** *Bacteroides intestinalis*, **(D)** *Bifidobacterium pseudolongum*, **(E)** *Parasutterella excrementihominis*, and **(F)** *Turicimonas muris*.

These results were generally consistent with LEfSe analyses (*strict all-vs.-all* setting; Figure 3), *B. dorei* is enriched in ObMet (Figures 3A,B) while *B. intestinalis* is enriched in the ObC (Figures 3A,C), with other taxa also enriched in the control group (Figures 3A,D–F).

### Contributional Pathway Diversity

**FIGURE 4:**
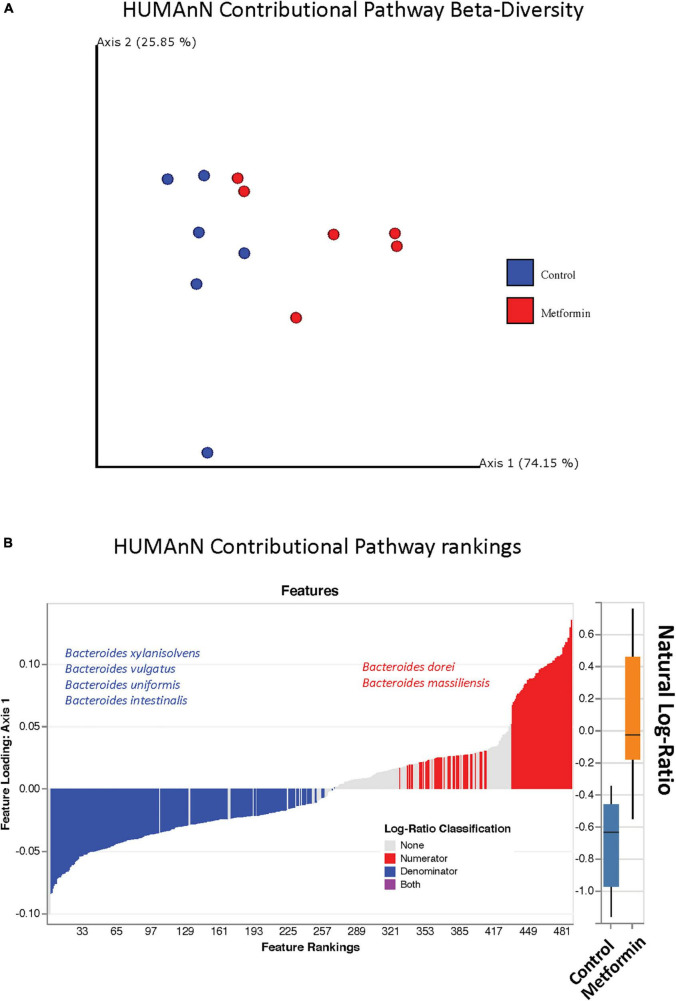
**(A)** Robust Aitchison PCA plot of metagenome samples processed through HUMAnN and DEICODE and visualized with qurro. Pathways not present in at least 50% of samples were removed from the analysis. These values Separation of Control and Metformin treated groups was significant (*p*-value 0.012). **(B)** Corresponding log-ratio rankings for Pathways oriented to Axis 1 (right), displaying only pathways contributed by *Bacteroides* spp. Numerator (red)/Denominator (blue) ratios of these pathways were computed to generate a box-whisker plot (left), *p*-value 0.008. These results were corroborated with songbird analyses.

**FIGURE 5:**
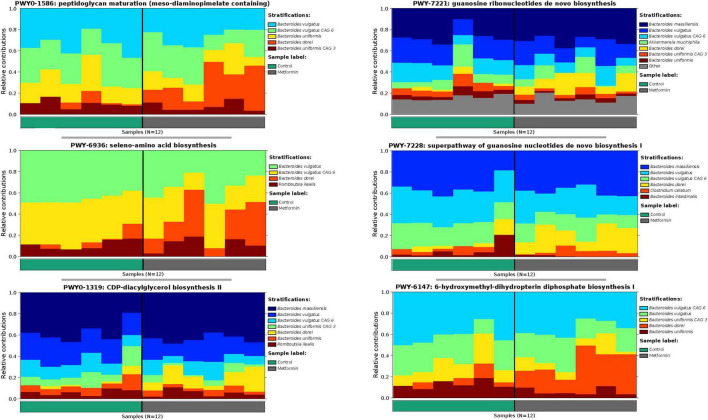
Differential contributional pathway diversity of top-ranked pathways, determined by DEICODE and qurro from HUMAnN analysis.

The observed contributional diversity of microbial taxa was also significantly associated with the treatment group (*p* < 0.012), as observed through unsupervised analysis *via* DEICODE (Figure 4A). The majority of all pathways were contributed by *Bacteroides* (Figure 4B). Although identical pathways can be contributed by all the *Bacteroides*, the specific species from which they are contributed, differs between the metformin and control groups. This can be exemplified through the differential contributional pathway diversity plots generated by HUMAnN, using some of the top-ranked pathways as determined by DEICODE (Figure 5 and Supplementary Figure 4). These results were confirmed using the supervised approach of Songbird (Supplementary Figure 5).

## Discussion

In this study, we examined the role of metformin, an anti-hyperglycemic agent, on the gut microbiome of obese female Zucker rats (*fa/fa*) during short-term treatment. This animal model is the most widely used model for non-diabetic obesity related research, unlike the Zucker Diabetic Fatty rat model. The primary cause for obesity in Zucker (*fa/fa*) rats is due to the mutation in the leptin receptor gene (fa) which is inherited by the rats as an autosomal recessive trait. The rats become noticeably obese by the age of 3–5 weeks, and by 14 weeks, >40% of their body is composed of lipids (Zucker, 1972; Bray et al., 1989). Furthermore, clinical studies and murine model systems have shown that short-term metformin treatment was sufficient to reduce liver steatosis (Lin et al., 2000; Madsen et al., 2015; Wang et al., 2016; Hakkak et al., 2021). Zucker rat models have also significantly contributed to the study of the function and role of microbiota in the gastrointestinal tract and its association with diseases such as metabolic disorder besides obesity (Gawler et al., 1989; Hakkak et al., 2017; Sui et al., 2019).

It is well established that the gut microbiota co-evolves with the host and the modified gut microbiome has been strongly linked with host obesity and associated therapeutics (Devaraj et al., 2013; Harsch and Konturek, 2018; Whang et al., 2019). Studies have shown that a high-fat diet for obesity, facilitates metabolic disorders, like Type 2 diabetes mellitus (T2DM) which may alter the gut microbiome (Pascale et al., 2019; Zhang and Hu, 2020). We reported the effects of obesity on the gut microbiome of Zucker rats using amplicon sequencing of the 16S rRNA gene. We found that obese rats showed higher ratios of the *Firmicutes* to *Bacteroidetes* phyla than the lean rats within a given timeframe, even though the rats were fed same high-fat diet. Gut microbiota populations appeared to shift with both the time point and observed phenotype (Hakkak et al., 2017).

Metformin is primarily administered alone or in combination with other hypoglycemic drugs to effectively control the blood glucose level of diabetic individuals, particularly to obese or overweight patients (Maruthur et al., 2016; Thomas and Gregg, 2017). Subsequently, evidence from multiple studies underscores the ability of metformin to reshape the gut microbiome in rat models induced by high-fat diet and in T2DM individuals (Zhang et al., 2015; Zhang and Hu, 2020). One of the studies reported that metformin exerts hypoglycemic effects by affecting the gut microbiome, which maintains intestinal barrier function, increases the production of short chain fatty acids, regulates bile acid metabolism, and affects glucose homeostasis. Hence, it is important to understand the relationships between obesity and the intestinal microbiota in correlation with the effects of metformin in obese Zucker rats significantly in their widespread use as a model disease system (Zhang et al., 2015).

Our study herein, PCA plots of beta-diversity showed the separation of each test group (ObC and ObMet) into different bacterial populations by unsupervised clustering through Aitchison distances. The dominating taxa that are associated with the separation of the metagenomic samples include *Bacteroides*, *Akkermansia*, *Bifidobacterium* (Figure 1). Previous studies related to metformin treatment on high-fat diet induced obese rats have also reported the alterations of similar taxa, particularly *Bacteroides*, *Bifidobacterium* (Zhang et al., 2019; Zhang and Hu, 2020). In one such study, Zhang and Hu (2020) has shown that metformin modulates the gut microbiota by enriching short chain fatty acid (SCFA) producing bacteria, like *Bacteroides* spp. We’ve observed similar taxonomic alterations through the analysis of log-ratios, based on MetaPhlAn output. Not only are *Bacteroides* spp. enriched HFD Zucker rats in general, but our shotgun metagenomics analysis revealed differences in the *Bacteroides* spp. that are differentially enriched between the treatment groups. For example, we observed that *B. dorei*, and *B. massiliensis* are enriched in ObMet, while *B. vulgatus*, *B. uniformis*, *B. intestinalis*, and *B. xylanisolvens* are enriched in ObC. The enrichment of *D. dorei* in the metformin-treated Zucker rats was also observed in rats with T2D (Yoshida et al., 2018; Zhang et al., 2020). Other studies in murine and human models often observe the co-enrichment of *B. vulgatus* and *B. dorei* across various disease states (Yoshida et al., 2018). However, we observe that *B. dorei* is enriched in ObMet while *B. vulgatus* is enriched in ObC (Figure 2 and Supplementary Figure 3). This pattern, where the ratio of *B. dorei* to *B. vulgatus* differs with respect to treatment groups, was also observed in blockade treatments in metastatic melanoma (Usyk et al., 2021) and with host epigenomic alterations of inflammatory bowel disease (Ryan et al., 2020). It was proposed by Ryan et al. (2020), that this pattern may be indicative of colonic-crypt species-specific colonization and competition (Sonnenburg et al., 2010; Lee et al., 2013) due to differences in metabolic capabilities (Gutiérrez and Garrido, 2019). Whether this, or other similar phenomenon, is occurring in our study system remains to be determined.

The most abundant bacteria reported in our study is *Bacteroides dorei*. Which is capable of metabolizing many drugs (Zimmermann et al., 2019), and could potentially be doing so with metformin. LEfSe analysis (Segata et al., 2011), also confirmed that *Bacteroides dorei* was enriched in metformin treated obese rats compared to the control group (Figure 3). *Bacteroides intestinalis*, *Bifidobacterium pseudolongum, Parasutterella excrementihominis*, and *Turicimonas muris* were found to be abundant in non-metformin treated obese rats.

A subsequent pathway ranking analysis revealed similar results about the taxa contributing functional pathways. The *Bacteroides* sp. described above were found to be contributing to the pathways from both test groups. Some taxa are reportedly found exclusively in mouse models like *Turicimonas* sp. (Lagkouvardos et al., 2016), while others may be present in both rat models and humans.

Recent evidence has shown that individuals who are genetically susceptible to various autoimmune diseases, like T2DM have substantial differences in gut microbial composition than non-genetically susceptible individuals (Bakir et al., 2006). Davis-Richardson et al. (2014) reported in their study about the role of *Bacteroides dorei* dominating the gut microbiome in Finnish children likely susceptible to type 1 diabetes mellitus (T1D). Significantly higher composition of *B. dorei* and *B. vulgatus* was found in the autoimmune susceptible children than in the control group with the help of metagenomic sequencing. The study also suggested the potential involvement of *B. dorei* in autoimmune disorders like T1D.

*Bacteroides vulgatus* has been associated with the reduced risk of immune-related adverse events (Usyk et al., 2021), including dermatological skin toxicity (where *B. dorei* anti-correlated) (Zhang et al., 2021). Furthermore, *B. vulgatus* has been shown to be protective against *Escherichia coli* induced colitis of interleukin-2-deficient gnotobiotic mice (Waidmann et al., 2003). In contrast, several studies revealed that *B. vulgatus* is generally viewed as a pathobiont (Ó Cuív et al., 2017), often associated with colitis in murine (Bloom et al., 2011) model systems, as well as human ulcerative colitis (Mills et al., 2022), irritable bowel disease (Ryan et al., 2020), and celiac disease (Schippa et al., 2010; Bloom et al., 2011; Wu et al., 2021). It has also been shown that *B. vulgatus* has an increased protease activity compared to other *Bacteroides* spp., and can cause barrier dysfunction (Riepe et al., 1980; Mills et al., 2022). We are unable to determine if this is at all related to the observed patterns described herein. This highlights the importance of ecological context when considering where a particular microbe lies on the “parasitism (pathogen)–mutualism” spectrum (Drew et al., 2021).

The major differences in this current study with previously related studies is the reliance of obese animals fed with same diets compared to high-fat diet for inducing obesity (Zhang et al., 2015). Also, the emphasis of metformin treatment and subsequent examination of the gut microbiota for compositional changes is less known in the scientific community. Thereby, the relationship between obesity, gut microbiome and the role of *B. dorei* demands further investigation in the Zucker rat model. Sample size is a major limiting factor in this study, a larger sample size applying short-term metformin treatment may strengthen the weight/power of the data.

Subsequently, the development of different chronic diseases is linked to obesity and also the host’s gut microbiome, where the relationship between the two plays a vital role in development of autoimmune diseases, cardiovascular disease, kidney disease and certain types of cancers (Wang et al., 2017; Vázquez-Baeza et al., 2018; Hobby et al., 2019; Kazemian et al., 2020; Sepich-Poore et al., 2021). Consequently, future investigations may be conducted on the relationship between obesity, metformin or other related drug, gut microbiota and several related diseases can be based on the standard data from provided by our study on the interaction and role of metformin correlated with obesity and the gut microbiome in Zucker rats.

## Data Availability Statement

The datasets presented in this study can be found in online repositories. The names of the repository/repositories and accession number(s) can be found below: https://www.ncbi.nlm.nih.gov/genbank/, PRJNA770726.

## Ethics Statement

The animal study was reviewed and approved by the Association for Assessment and Accreditation of Laboratory Animal Care approved animal facility. This facility is registered with the USDA and has a fully approved Letter of Assurance on file with the Office of Laboratory Animal Welfare of the National Institutes of Health.

## Author Contributions

RH conceived of the project, acquired the funding, and conducted laboratory experiments. CR, RH, and SB collected the data. MR analyzed the data. All authors contributed to the writing of the manuscript.

## Conflict of Interest

The authors declare that the research was conducted in the absence of any commercial or financial relationships that could be construed as a potential conflict of interest.

## Publisher’s Note

All claims expressed in this article are solely those of the authors and do not necessarily represent those of their affiliated organizations, or those of the publisher, the editors and the reviewers. Any product that may be evaluated in this article, or claim that may be made by its manufacturer, is not guaranteed or endorsed by the publisher.

## Supplementary Material

Supplementary Figure 1 Overall study design.

Supplementary Figure 2 Metagenomics workflow.

Supplementary Figure 3 Songbird differential rankings processed through MetaPhlAn. **(A)** Same plot as in Figure 1B with all features above and below 0 selected, and their ratios plotted as a box-whisker-plot (right). **(B)** Same plot as with Figure 2A, with only the top-feature rankings selected (left), and their ratios plotted as a box-whisker-plot (right). **(C)** Same as Figure 2B, only *Bacteroides* spp. selected (left) and their ratios plotted as box-whisker plot (right).

Supplementary Figure 4 Songbird differential rankings of HUMAnN pathways. These are shown in the HUMAnN contributional diversity plot of Figure 5.

Supplementary Figure 5 Songbird differential rankings processed through HUMAnN. **(A)** Same plot as in Supplementary Figure 3A with all features above and below 0 selected, and their ratios plotted as a box-whisker-plot (right). **(B)** Same plot as with Supplementary Figure 3B, with only the top-feature rankings selected (left), and their ratios plotted as a box-whisker-plot (right). **(C)** Same as Supplementary Figure 3C, only *Bacteroides* spp. selected (left) and their ratios plotted as box-whisker plot (right).
