# Supplementary figures and images for: Short-Term Metformin Treatment Enriches Bacteroides dorei in an Obese Liver Steatosis Zucker Rat Model
Source: Front Microbiol. 2022 Mar 30;13:834776. doi: 10.3389/fmicb.2022.834776 (PMC9006818; doi:10.3389/fmicb.2022.834776)

## Study Design

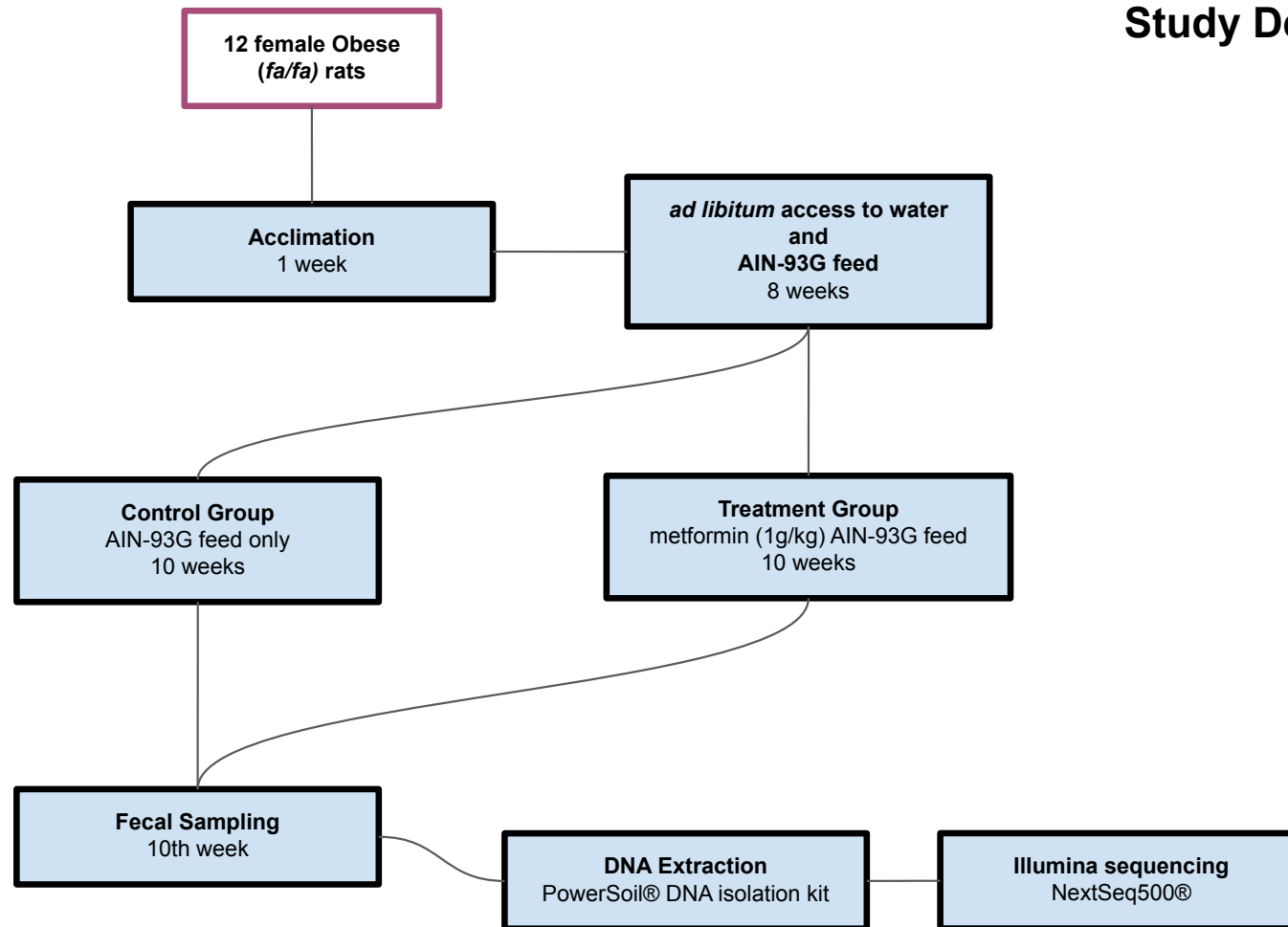

Supplement: Supplementary Figure 1 — Overall study design. [file Image_1.pdf]

Metagenomics Pipeline

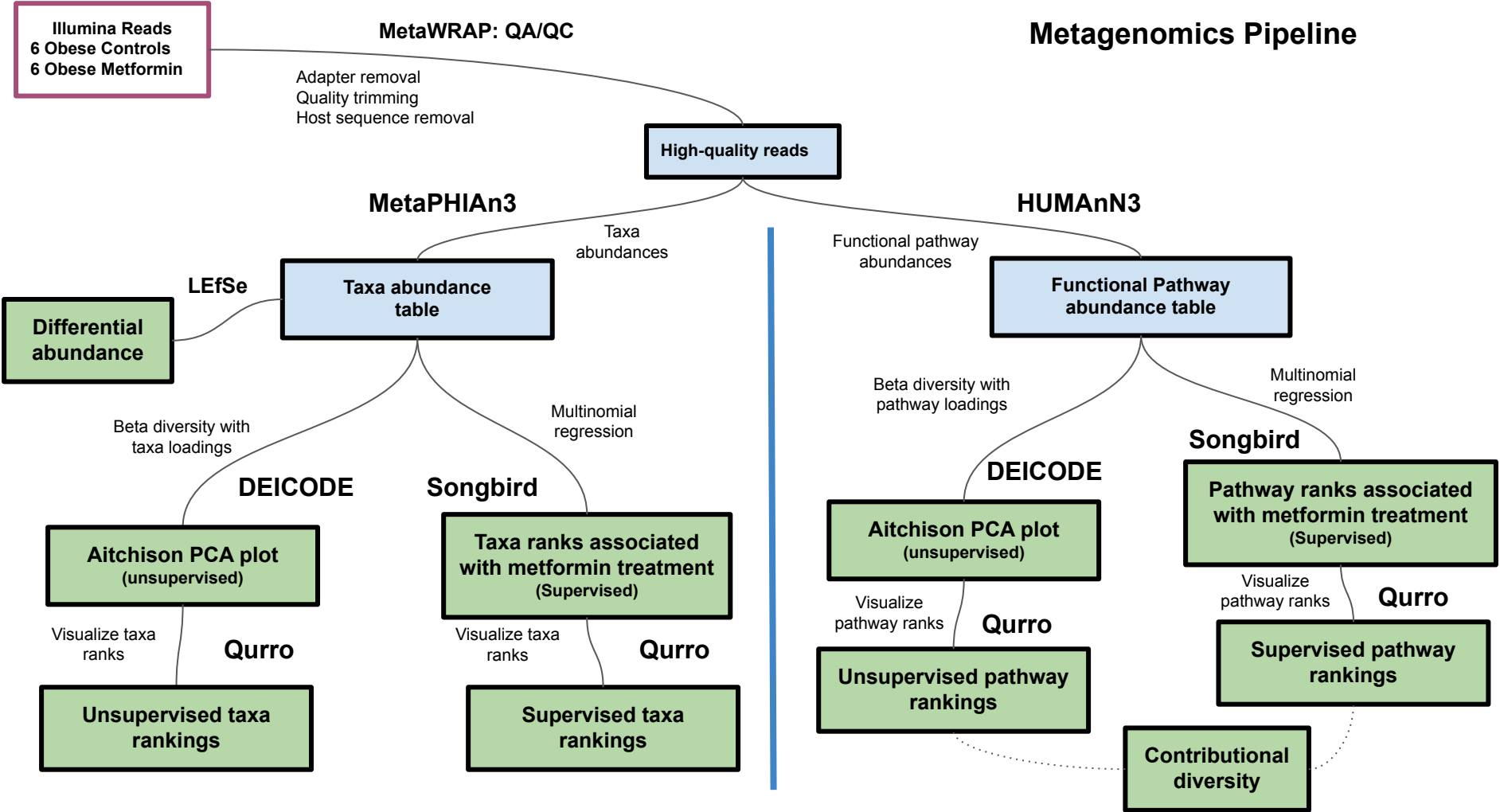

Supplement: Supplementary Figure 2 — Metagenomics workflow. [file Image_2.pdf]

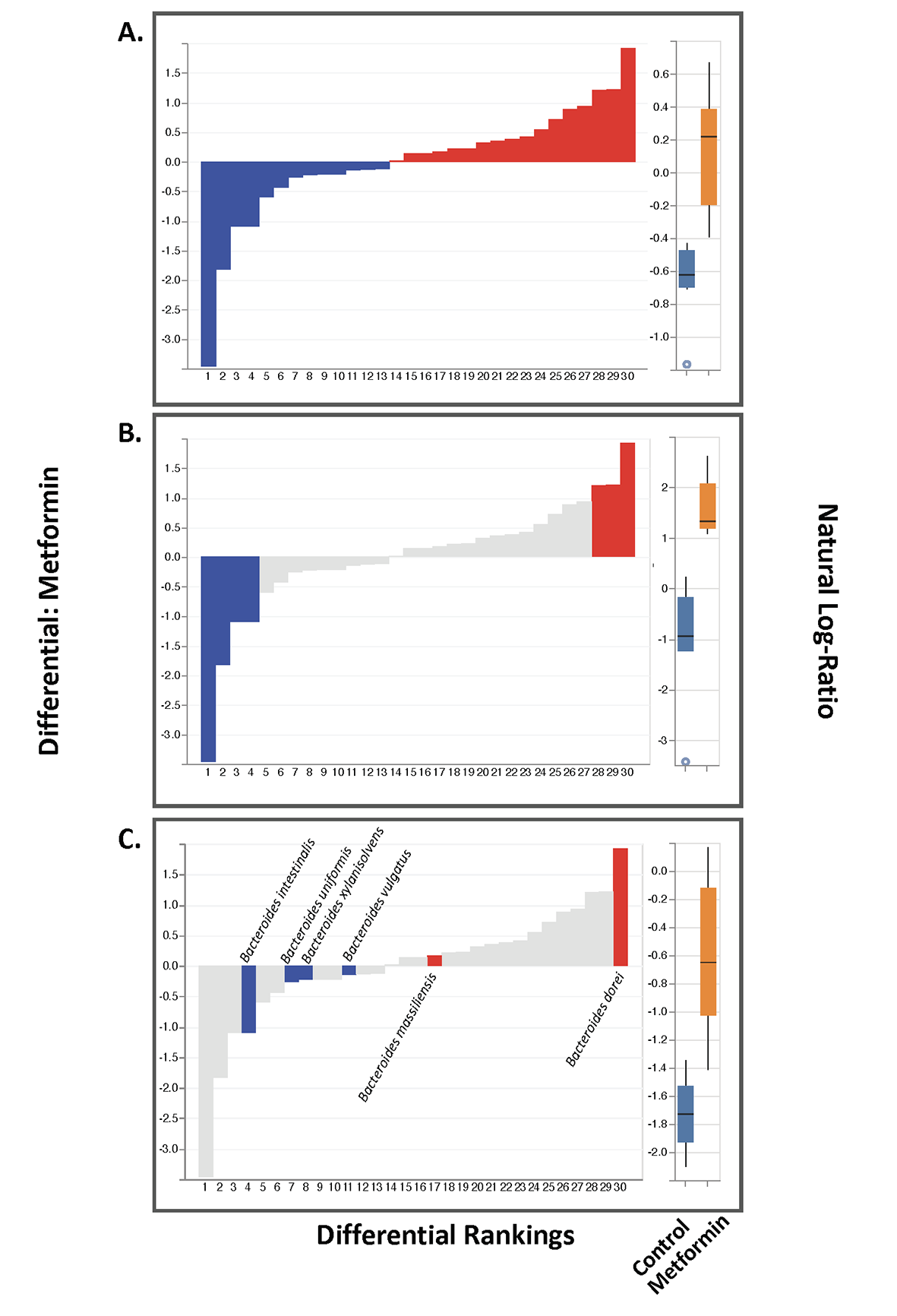

Supplement: Supplementary Figure 3 — Songbird differential rankings processed through MetaPhlAn. (A) Same plot as in Figure 1B with all features above and below 0 selected, and their ratios plotted as a box-whisker-plot (right). (B) Same plot as with Figure 2A, with only the top-feature rankings selected (left), and their ratios plotted as a box-whisker-plot (right). (C) Same as Figure 2B, only Bacteroides spp. selected (left) and their ratios plotted as box-whisker plot (right). [file Image_3.tiff]

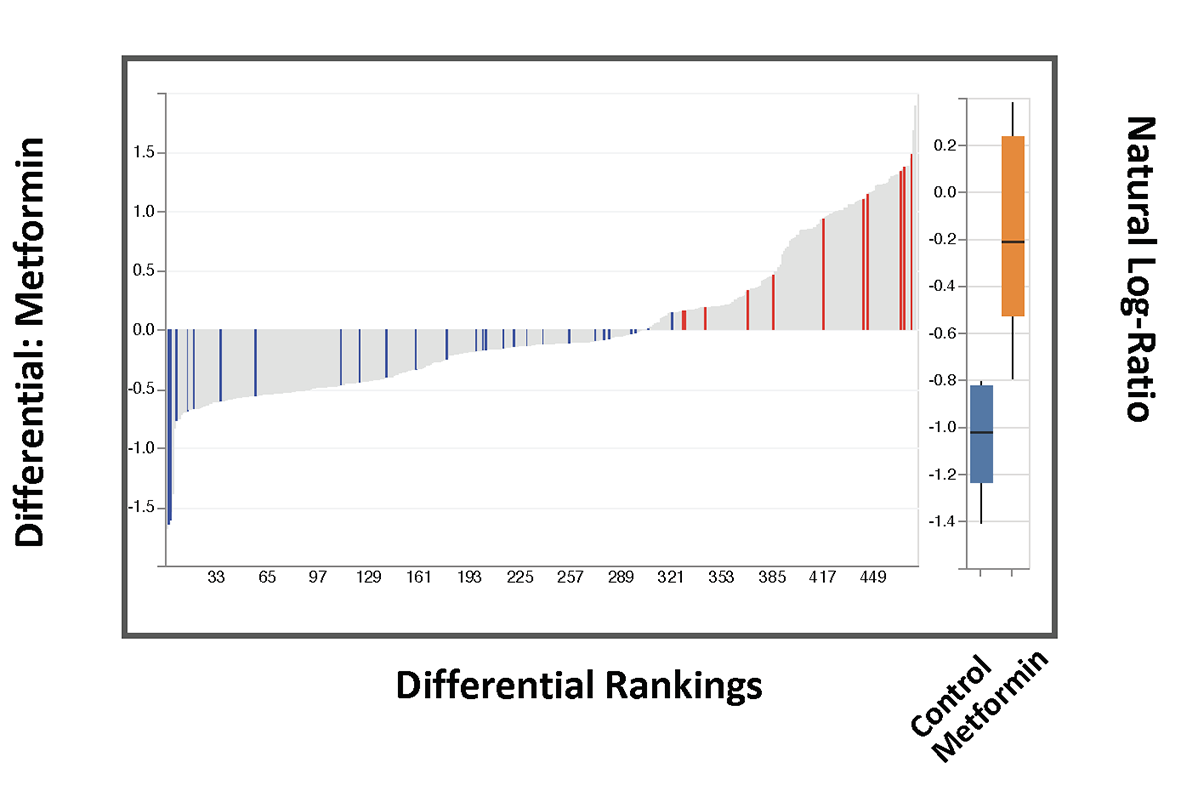

Supplement: Supplementary Figure 4 — Songbird differential rankings of HUMAnN pathways. These are shown in the HUMAnN contributional diversity plot of Figure 5. [file Image_4.tiff]

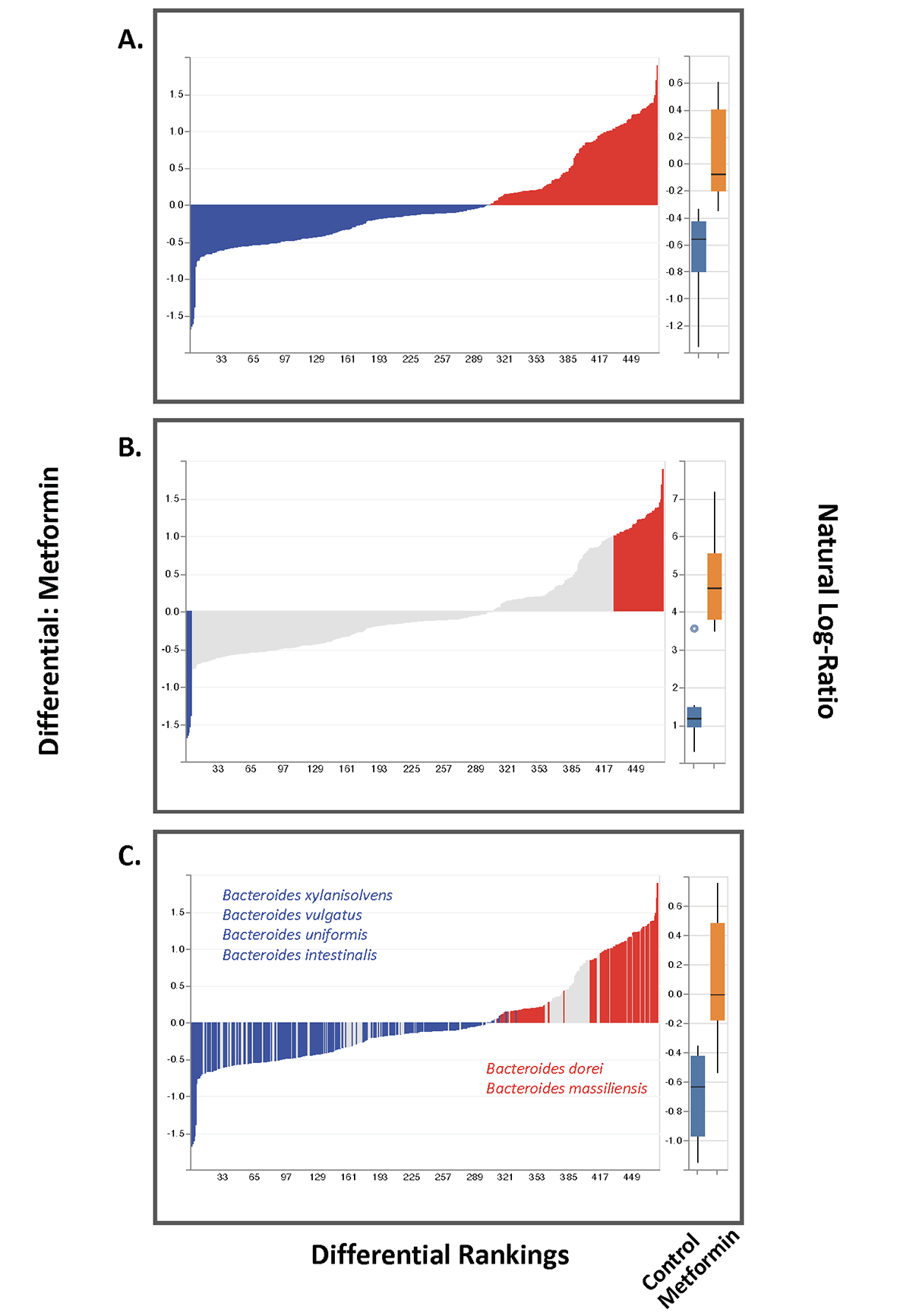

Supplement: Supplementary Figure 5 — Songbird differential rankings processed through HUMAnN. (A) Same plot as in Supplementary Figure 3A with all features above and below 0 selected, and their ratios plotted as a box-whisker-plot (right). (B) Same plot as with Supplementary Figure 3B, with only the top-feature rankings selected (left), and their ratios plotted as a box-whisker-plot (right). (C) Same as Supplementary Figure 3C, only Bacteroides spp. selected (left) and their ratios plotted as box-whisker plot (right). [file Image_5.tiff]
